# Supplementary material for: Time-Resolved Profiling Reveals ATF3 as a Novel Mediator of Endocrine Resistance in Breast Cancer
Source: Cancers (Basel). 2020 Oct 11;12(10):2918. doi: 10.3390/cancers12102918 (PMC7650760; doi:10.3390/cancers12102918)
Supplement: Supplementary file 1 [file cancers-12-02918-s001.zip › Supplementary figures.pdf]

cancers
MDPI

Supplementary Materials

Time-Resolved Profiling Reveals ATF3 as a Novel Mediator of Endocrine Resistance in Breast Cancer

Simone Borgoni, Emre Sofyalı, Maryam Soleimani, Heike Wilhelm, Karin Müller-Decker, Rainer Will, Ashish Noronha, Lukas Beumers, Pernette J. Verschure, Yosef Yarden, Luca Magnani, Antoine H.C. van Kampen, Perry D. Moerland and Stefan Wiemann

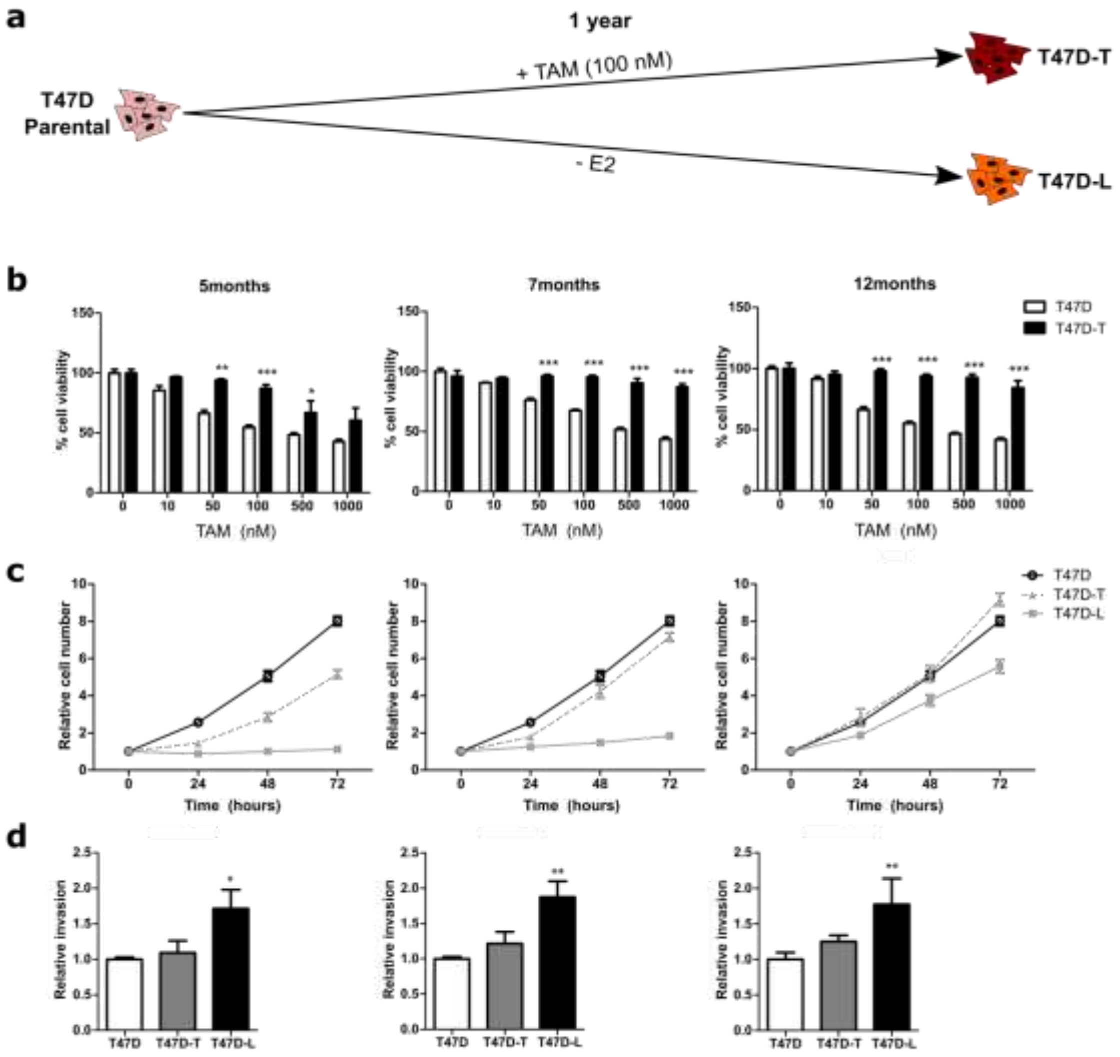

Figure S1. Generation and characterization of resistance development in T47D
(a) Schematic representation of resistance development during 1 year of treatment with 100nM TAM (T47D-T) or E2 deprivation (T47D-L). (b) Viability of cells treated for 72h with increasing doses of TAM. Assay measurement performed with CellTiterGlo at indicated time points during resistance development. (c) Nuclei count at indicated time points during resistance development. (d) 72h transwell invasion assay measured at indicated time points during resistance development. Data are represented as relative values normalized to the control. Values are represented as mean ± SD, n=3. *** p-value <0.001, ** p-value <0.01, * p-value <0.05

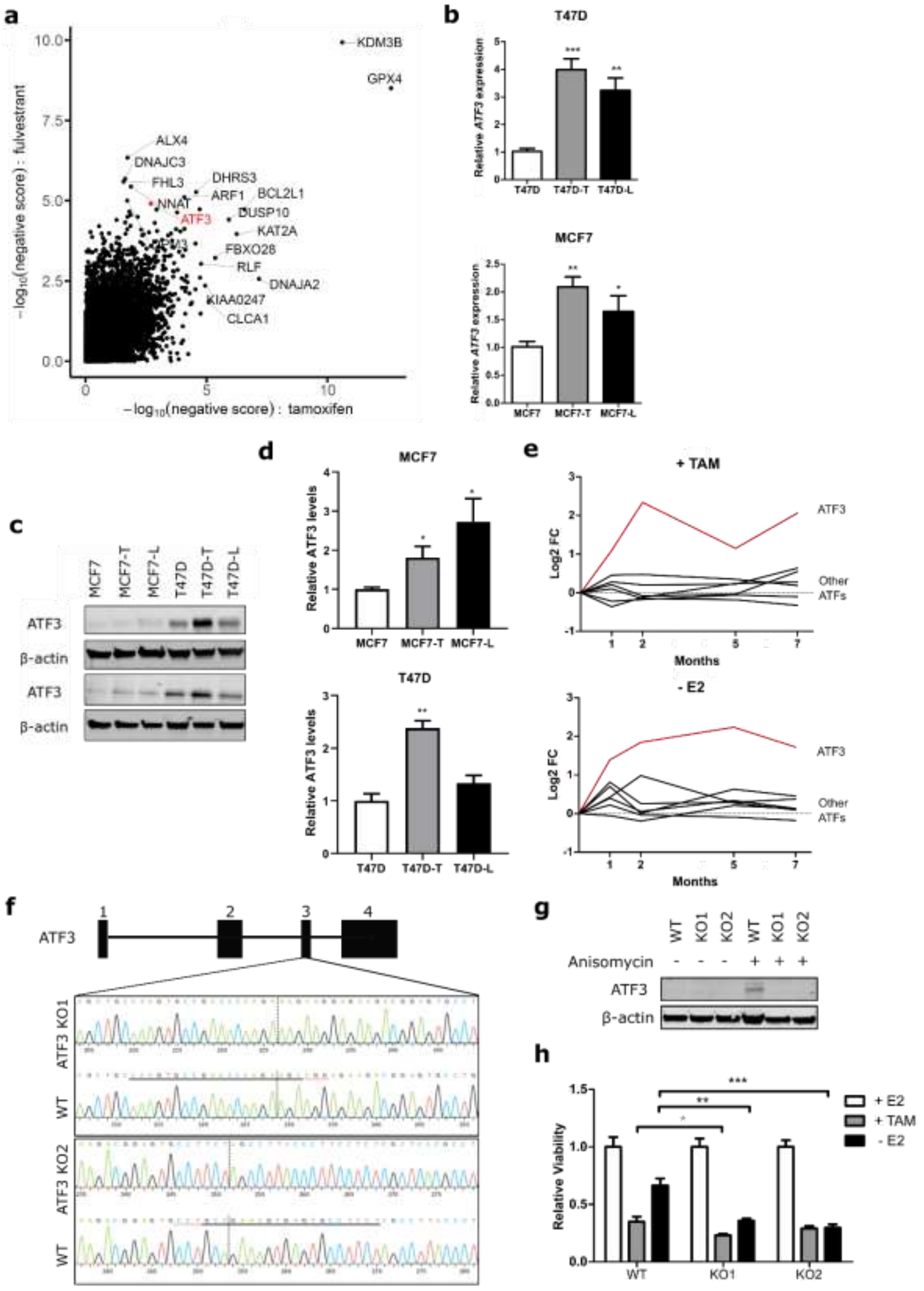

Figure S2. Crispr/Cas9 knockout of ATF3 in MCF7 cells
(a) CRISPR screen from Nagarajan et al., Nat Genet 2020; 52:187-197. Negative score represents gRNA depletion (genes promoting drug resistance). Genes that are in the top-10 of tamoxifen or fulvestrant negative score are labeled. (b) Relative ATF3 mRNA levels determined by qRT-PCR in T47D and MCF7

sensitive and resistant cells. (c) ATF3 protein levels in sensitive and resistant T47D and MCF7 determined by WB after 2h anisomycin stimulation. β-actin levels are used as loading control. (d) Bands intensity quantification of ATF3 levels in sensitive and resistant T47D and MCF7. Data are normalized to β-actin and reported as Fold Change compared to parental cells. (e) RNA-seq values represented as relative Log2 Fold Change compared to parental cells of ATF family members during resistance development. (df) Gene scheme and Sanger sequencing tracks visualization of alterations in exon 3 of the ATF3 gene that were induced by sgRNA-mediated CRISPR/Cas9 knockout, compared to the wildtype (WT) sequence. Sequences of respective sgRNAs are underlined (above the WT sequence track) and PAM-sequences (AGG) are indicated with a red dotted line (https://ice.synthego.com). (eg) Western blot validation of ATF3 knockout efficiency with or without anisomycin stimulation. β-actin is used as loading control. (fh) Cell viability of MCF7 after 8 days of treatment with E2, TAM or without E2 measured with CellTiterGlo. All values are normalized to the respective cell treated with E2. Data are represented as mean ± SEM, n=3. *** p-value <0.001, ** p-value <0.01, * p-value <0.05

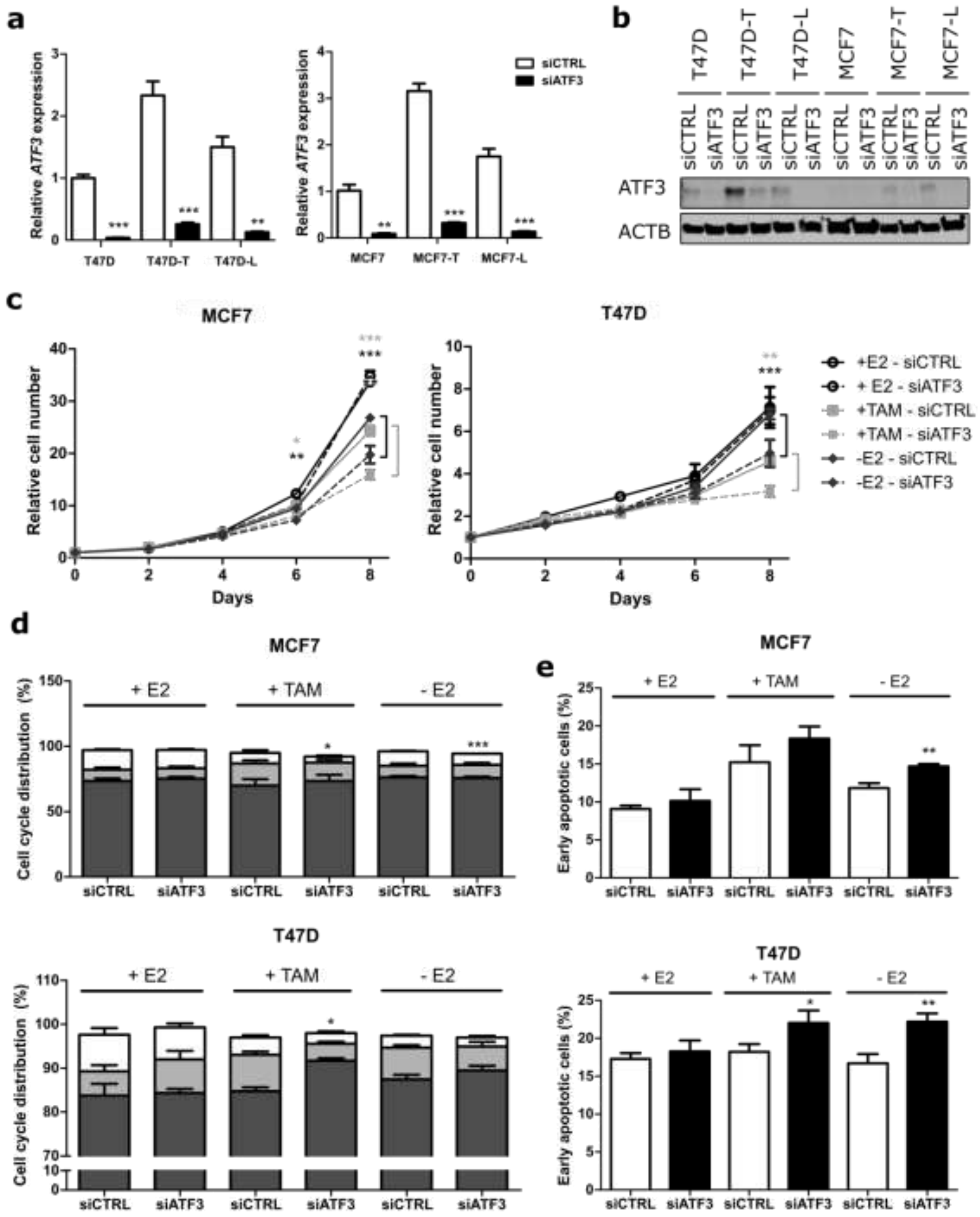

Figure S3. ATF3 knockdown
(a) ATF3 mRNA levels determined by qRT-PCR 3 days after transfection with siCTRL and siATF3. Values are normalized to ACTB levels and to the siCTRL. (b) ATF3 protein levels determined by WB 3 days after transfection with siCTRL and siATF3 and 2h stimulation with anisomycin. β-actin levels are used as loading control. (c) Cell proliferation of MCF7 and T47D cells treated for 8 days after transfection with siCTRL or siATF3 with E2, TAM or deprived from E2 and measured at indicated time points with nuclei count in fluorescent microscopy. All values are normalized to a seeding control. (d) Cell cycle distribution of MCF7 and T47D cells treated for 4 days after transfection with siCTRL or siATF3 with E2, TAM or without E2. Plots represent the percentage of cells in the different cell cycle phases determined by BrdU/7AAD staining. Statistics performed on the S phases (white bars) (e) Measurement of apoptosis rate in MCF7 and T47D cells treated for 4 days after transfection with

siCTRL or siATF3 with E2, TAM and without E2. Plots represent the percentage of early apoptotic cells determined by Annexin V/PI staining. Data are represented as mean ± SEM, n=3. *** p-value <0.001, ** p-value <0.01, * p-value <0.05

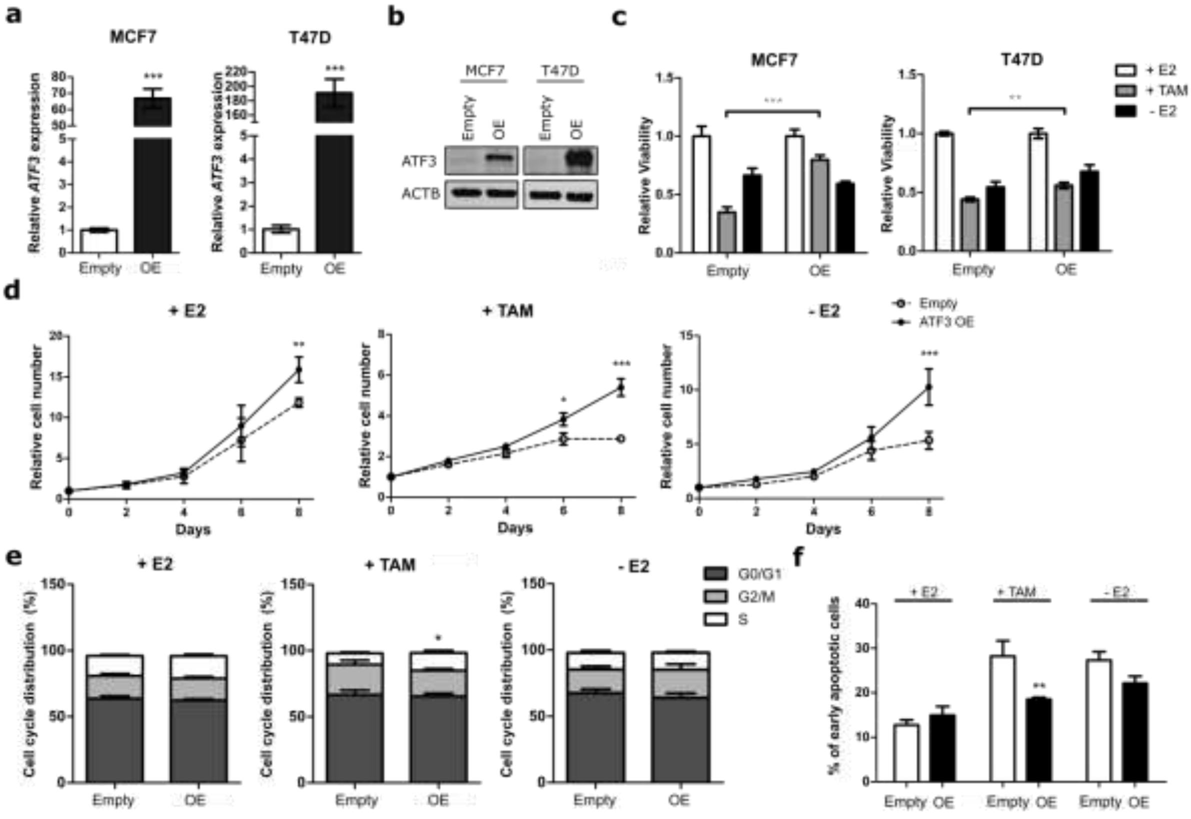

Figure S4. ATF3 overexpression reduces sensitivity to endocrine therapy in T47D cells

(a) ATF3 mRNA levels determined by qRT-PCR in MCF7 and T47D cells carrying either the empty vector or the ATF3 overexpressing vector. (b) Western blot validation of overexpression efficiency. β-actin is used as loading control. (c) Cell viability of MCF7 and T47D after 8 days of treatment with E2, TAM or E2 deprivation measured with CellTiterGlo. (d) Cell proliferation of MCF7 treated with E2, TAM or E2 deprivation for 8 days and measured at indicated time points with nuclei count in fluorescent microscopy. (e) Cell cycle distribution of MCF7 treated for 4 days with E2, TAM or E2 deprivation. Cell cycle distribution represented as percentage of cells in the different cell cycle phases determined by BrdU/7AAD staining. Statistics performed on the S phases (white bars). (f) Measurement of apoptosis rate in T47D treated for 4 days with E2, TAM or E2 deprivation. The plot represents the percentage of early apoptotic cells determined by Annexin V/PI staining. Data are represented as mean + SEM, n=2 or n=3. *** p-value <0.001, ** p-value <0.01, * p-value <0.05

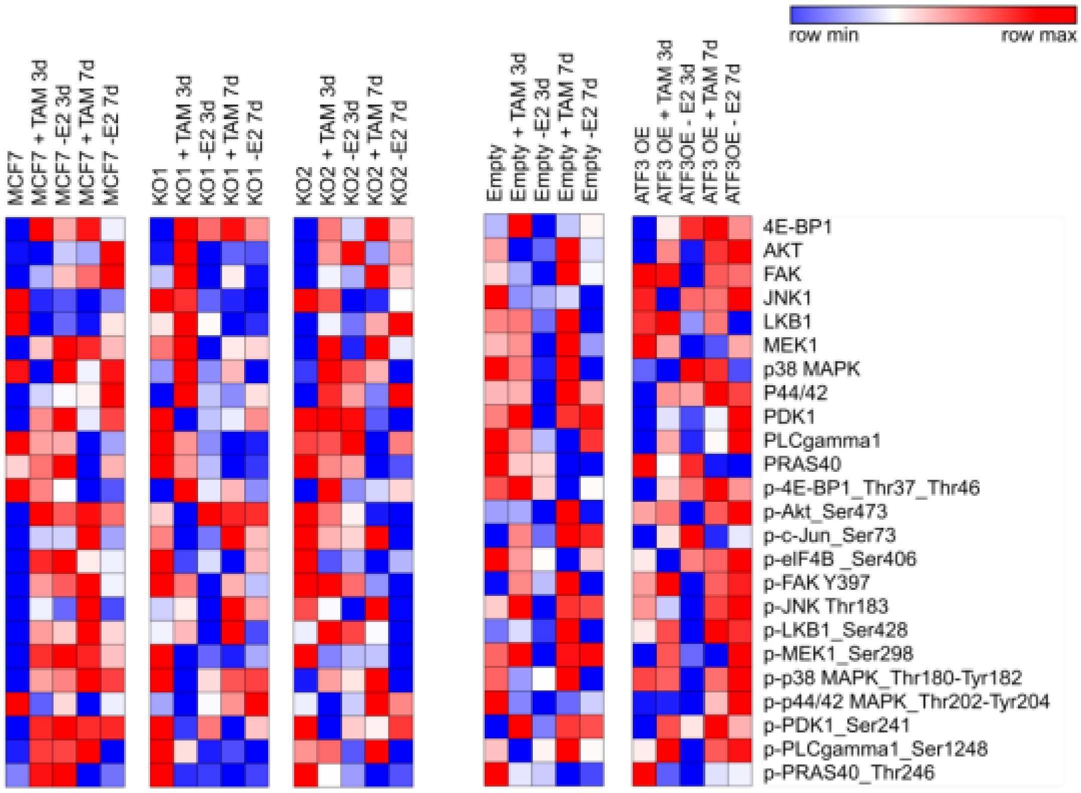

Figure S5. ATF3 regulates AKT/MAPK pathways activation upon endocrine therapy treatement. Heatmaps of 24 total and phospho-proteins involved in PI3K-AKT-MAPK signaling pathway in MCF7 cells treated for 3 or 7 days with either TAM or E2 deprivation. Log2 normalized signal intensities for each protein are plotted and color-coding refers to relative intensities in each row and each cell line independently.

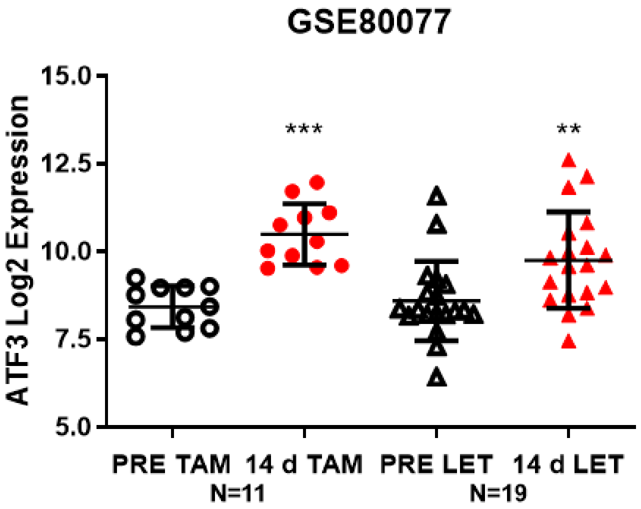

Figure S6. Separation of patients from GSE80077 in TAM-treated and LET-treated
ATF3 expression in GSE80077, comprised of matched tumors before (PRE) and after 14 days of neoadjuvant endocrine therapy administration. Patients are separated based on the type of endocrine therapy received. Values are represented as Log2 expression of the respective probe in individual patients ± SD. *** p-value <0.001, ** p-value <0.01, * p-value <0.05

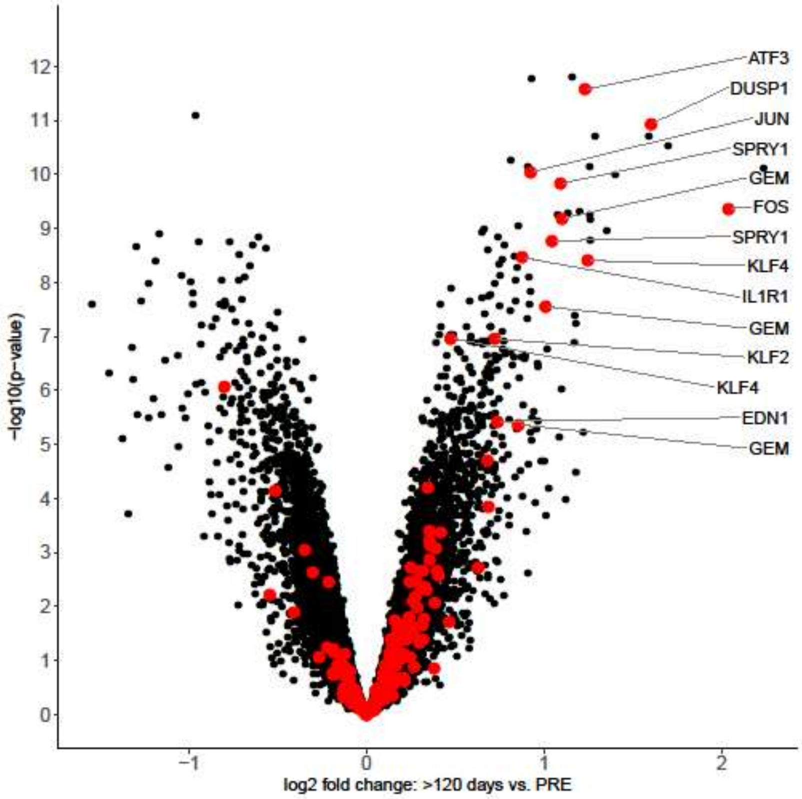

Figure S7. Early upregulated genes in GSE111563

Volcano plot of the early upregulated gene set (Fig. 1a-b) in the GSE111563 showing Log2 fold change between >120 days and pre-treatment time points versus p-value (-log10 scale). Red dots represent genes in the early upregulated gene set (Supplementary Table 1). Quantile normalized data for GS 111563 was retrieved from GEO using the GEOquery R/Bioconductor package. Gene-wise linear models were fitted using the limma R/Bioconductor package. A consensus intrapatient correlation was estimated for measurements on the same subject (function 'duplicateCorrelation', package limma) and included in the linear model fit. Differential gene expression between the different time points was assessed via a moderated t-test.
