# Supplementary figures and images for: Time-Resolved Profiling Reveals ATF3 as a Novel Mediator of Endocrine Resistance in Breast Cancer
Source: Cancers (Basel). 2020 Oct 11;12(10):2918. doi: 10.3390/cancers12102918 (PMC7650760; doi:10.3390/cancers12102918)

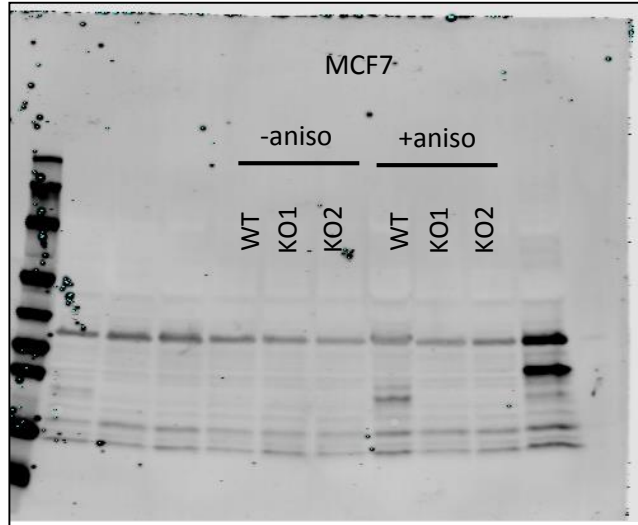

ATF3

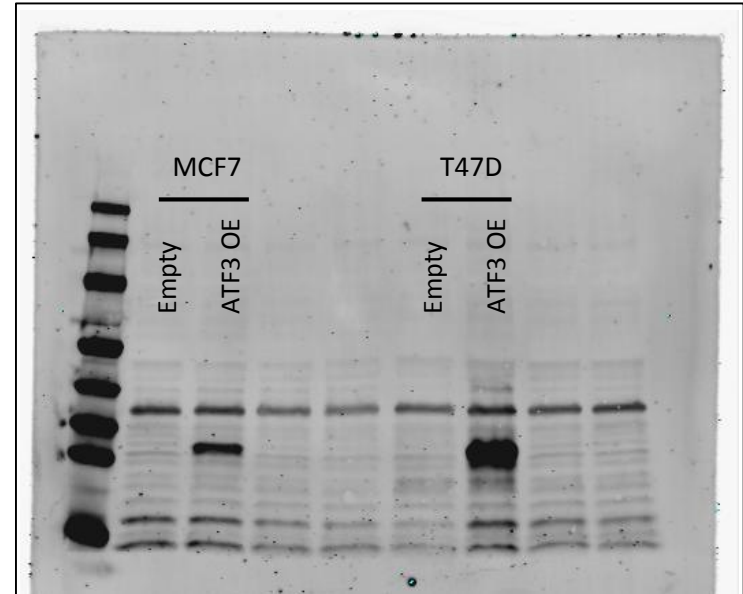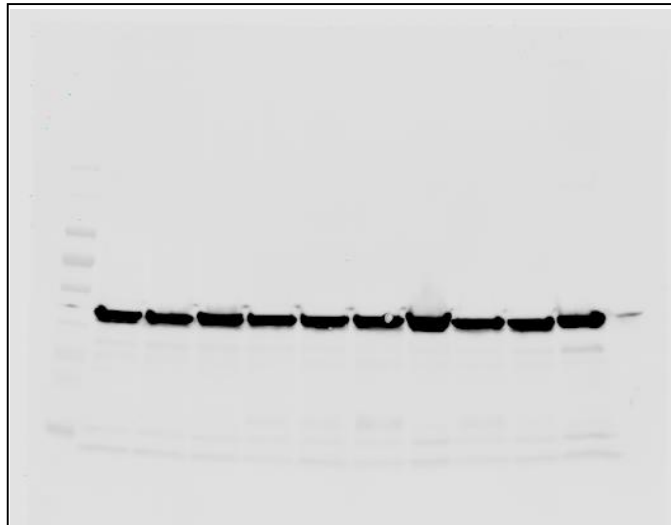

ACT

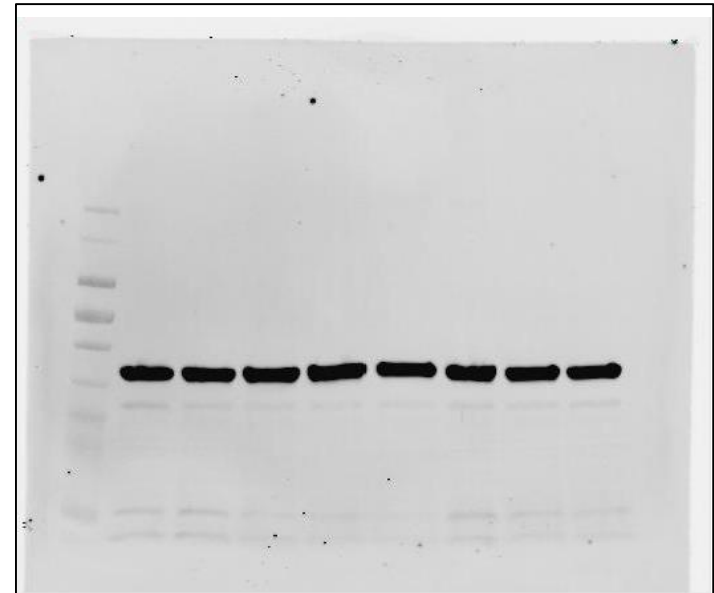

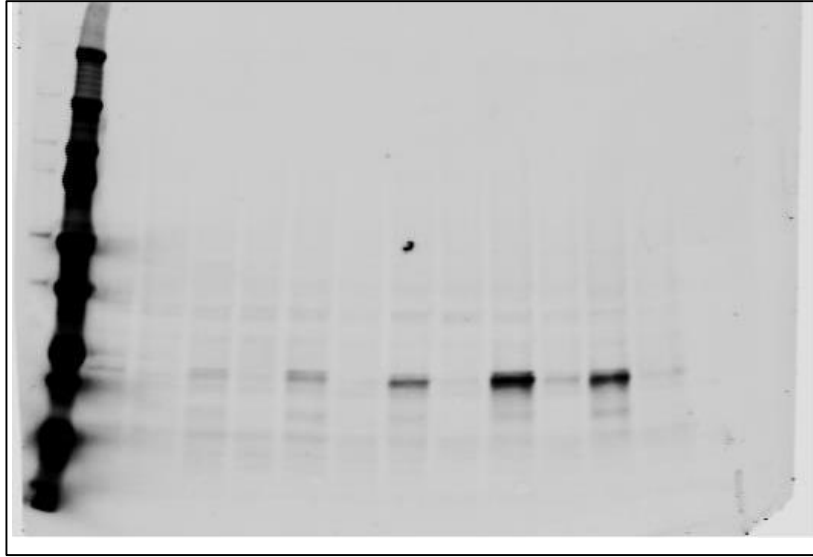

ATF3

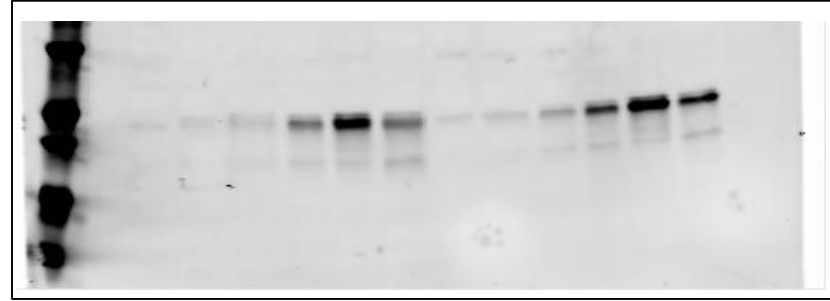

ACTB

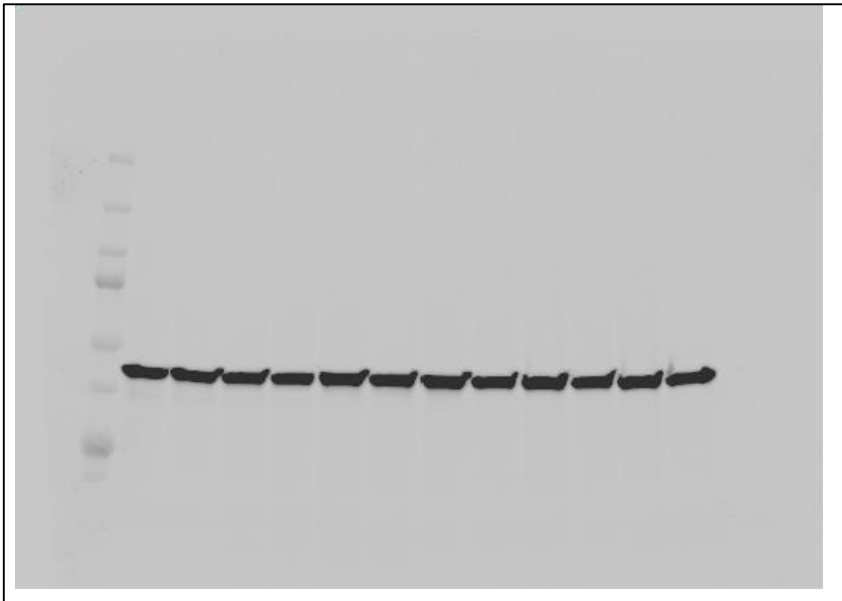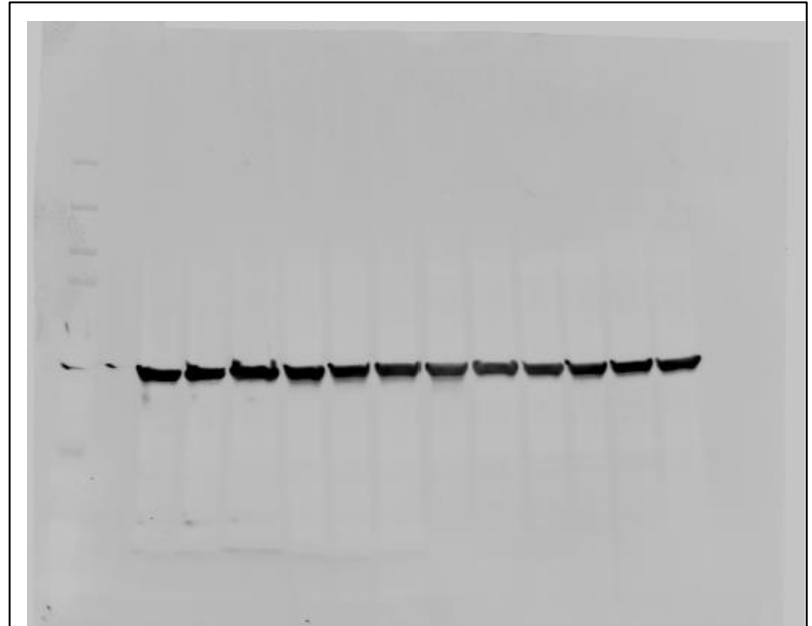

ATF3

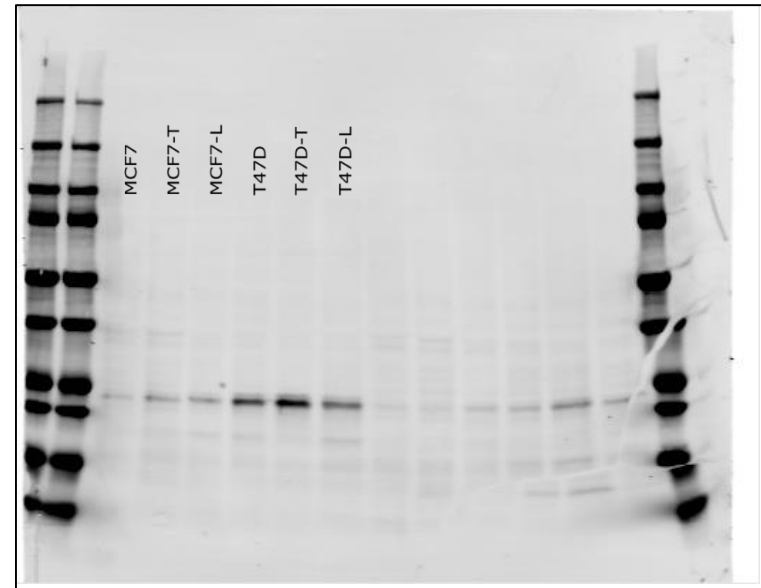

ACTB

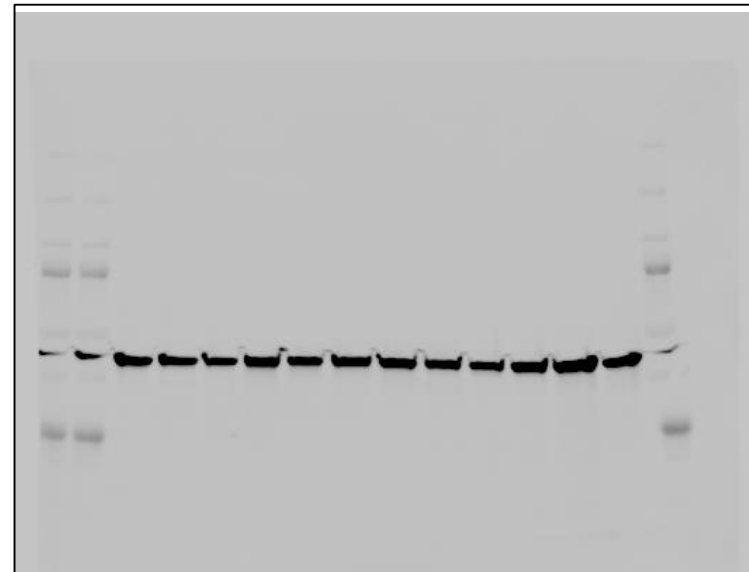

Supplement: Supplementary file 1 [file cancers-12-02918-s001.zip › western blots-For all Western blot figures, please mark molecular weight for the markers bands..pdf]
